# Retraction notice for: “PDK2 promotes chondrogenic differentiation of mesenchymal stem cells by upregulation of Sox6 and activation of JNK/MAPK/ERK pathway” [Braz J Med Biol Res (2017) 50(2): e5988]

**DOI:** 10.1590/1414-431X20205988retraction

**Published:** 2020-08-17

H. Wang^1*^, X.B. Shan^2*^, and Y.J. Qiao^3^

^1^Second Department of Orthopedics, Baodi Clinical College, Tianjin Medical University, Tianjin, China

^2^Second Department of Orthopedics, The First People’s Hospital of Yibin, Yibin, China

^3^First Department of Orthopedics, 4th (Xing Yuan) Hospital of Yulin, Yulin, China

Correspondence: Y.J. Qiao: <qiaoyongjun747@126.com>

*These authors contributed equally to this study.

Retraction: Braz J Med Biol Res | doi: http://10.1590/1414-431X20165988 | PMID: 28225870 | PMCID: PMC5343558

On July 15, 2020, the Brazilian Journal of Medical and Biological Research (BJMBR) received a request from the Corresponding author Yongjun Qiao to withdraw this manuscript: “*My research group is still conducting research on the effect of PDK2 on chondrogenic differentiation of mesenchymal stem cells. Some of the current experimental results are inconsistent with the previous results. Due to the need for further confirmation of the experimental results, in order not to mislead the readers, we request withdrawal*”.

Meanwhile, the Editors became aware of a denouncement published by independent journalists from the “For Better Science” website including this paper. This denouncement consisted of potential data falsification and/or inaccuracy of results in western blots and flow cytometry plots.

As per consensus between the Editors-in-Chief of the BJMBR and the Authors, this article has been retracted.

